# Atoms in separated resonators can jointly absorb a single photon

**DOI:** 10.1038/s41598-020-78299-x

**Published:** 2020-12-10

**Authors:** Luigi Garziano, Alessandro Ridolfo, Adam Miranowicz, Giuseppe Falci, Salvatore Savasta, Franco Nori

**Affiliations:** 1 Theoretical Quantum Physics Laboratory, RIKEN Cluster for Pioneering Research, Wako, Saitama 351-0198 Japan; 2 Dipartimento di Fisica e Astronomia, Università di Catania, 95123 Catania, Italy; 3 INFN Sezione Catania, Catania, Italy; 4 Faculty of Physics, Adam Mickiewicz University, 61-614 Poznan, Poland; 5 CNR-IMM Catania e INFN Sezione Catania, Catania, Italy; 6 Dipartimento MIFT, Università di Messina, 98166 Messina, Italy; 7 Department of Physics, University of Michigan, Ann Arbor, MI 48109-1040 USA

**Keywords:** Quantum physics, Single photons and quantum effects

## Abstract

The coherent nonlinear process where a single photon simultaneously excites two or more two-level systems (qubits) in a single-mode resonator has recently been theoretically predicted. Here we explore the case where the two qubits are placed in different resonators in an array of two or three weakly coupled resonators. Investigating different setups and excitation schemes, we show that this process can still occur with a probability approaching one under specific conditions. The obtained results provide interesting insights into subtle causality issues underlying the simultaneous excitation processes of qubits placed in different resonators.

## Introduction

For many decades the possibility of reaching the strong coupling regime between light and matter has been one of the major topics of research in atomic physics and quantum optics, driving the field of cavity quantum electrodynamics (QED). In this regime, which was first reached in Rydberg atoms interacting with the electromagnetic field confined in a high-Q cavity^1^, it is possible to observe a coherent and reversible energy exchange between light and matter, called vacuum Rabi oscillations, at a coupling rate exceeding the losses of the system. In 1992, the strong coupling regime was experimentally achieved^2^ also with single atoms coherently interacting with an optical cavity. Following these pioneering experiments, this strong regime of light–matter coupling has been realized in various quantum systems, enabling tests of fundamental physics and the study of single atom-photon processes^3^, and leading to important applications in quantum computation, quantum information processing, sensing and metrology^4–6^.

More than two decades after the observation of the strong coupling regime, the cavity-QED community started investigating the possibility of accessing a new non-perturbative light–matter regime in which the coupling rate can become a significant fraction of the bare energies of the system. In 2005, it was predicted^7^ that this new ultrastrong coupling (USC) regime could be observed with a planar microcavity photon mode which is strongly coupled to a semiconductor intersubband transition in the presence of a two-dimensional electron gas. In this new USC regime, the rotating-wave approximation (RWA) employed in the standard Jaynes–Cummings model^8,9^, which has been a workhorse of quantum optics in the weak and strong coupling regimes, cannot be safely applied anymore^10,11^. Indeed, it has been shown that the counter-rotating terms in the system Hamiltonian become relevant (see, e.g., Refs.^12–15^), giving rise to a wide variety of novel and unexpected physical phenomena^16–29^.

A proper description of the USC-regime physics requires to solve some fundamental theoretical issues, such as the failure of the usual normal-order correlation functions to describe the correct output photon emission rate^30–33^, some unphysical predictions of the standard master equation approach^34–36^, and gauge ambiguities in the quantum Rabi and Dicke models^37–39^. Besides the vast phenomenology that has been predicted to be observable in this new light–matter regime, the interest has also been fostered by the experimental realization of USC in several physical systems, including superconducting quantum circuits^40–52^, intersubband polaritons in microcavity-embedded doped quantum wells^7,53^, and other hybrid cavity-QED systems, such as Landau polaritons^54–58^, microcavity exciton polaritons^59–63^, magnons in microwave cavities^64,65^ and organic molecules^13,66–77^. Among unique physical effects of the USC regime, there are those related to the hybridization of the ground state of the quantum Rabi Hamiltonian^7,12,17,24,78–80^. Such a ground state now contains *virtual* excitations that can be released only by applying a time-dependent perturbation to the system. Moreover, the USC regime opens the possibility of observing higher-order processes and nonlinear optics with two-level systems and virtual photons^25,26,81^, symmetry breaking and Higgs mechanism^82^, multiphoton quantum Rabi oscillations^21^, and even more counterintuitive phenomena like the emission of bunched light from individual qubits^83^.

One of the most interesting nonlinear optical effects predicted in the USC regime consists of the simultaneously excitation of two or more spatially separated atoms by a single photon^23,84–86^. This last puzzling result, which has been studied in a quantum system constituted by two qubit ultrastrongly coupled to a single-mode resonator, provides new insights into the various quantum aspects of the interaction between light and matter and can find useful applications for the development of novel quantum technologies. Although this effect clearly demonstrates a relevant role of the counter-rotating terms and virtual processes in the USC regime, the single-mode approach does not allow to fully understand some subtle causality issues underlying this process, since the resonator mode is completely delocalized along the cavity. Specifically, a drawback of this simplified description of the electromagnetic field is that *any information about the spatial separation between the two atoms is lost*. Hence, the question arises if it is possible to observe this effect in the presence of natural or artificial atoms which are *actually* spatially separated. Here we provide a positive answer to this question, even though further work will be required for a full understanding of the impact of the spatial separation of the atoms on the joint absorption and emission of single photons. Moreover, it has been recently pointed out^87^ that the description of a cavity-QED system in terms of the single-mode quantum Rabi model in the USC and deep-strong coupling (DSC) regimes can lead to the violation of relativistic causality, and a multi-mode version of the quantum Rabi model is required in order to fully capture the propagation properties of the light field necessary to comply with causality. Here, although we do not adopt a multimode approach, we still assume the atoms to be spatially separated. Specifically, we introduce a simplified description of spatial separation, considering the two atoms embedded in different single-mode resonators. For example an array of *N* nearest-neighbour single-mode coupled resonators corresponds to a system with *N* distinct sites, like in tight-binding models in solid state physics, where propagation effects are taken into account. In these models the propagation speed depends on the interaction rate *J* between nearest neighbours cavities. For example the complete transfer of a photon from one resonator to the adjacent one requires a time $T = \pi / (2J)$.

In the present work, we show that the simultaneous excitation process of two qubits by a single cavity photon, as described in Ref.^23^, can take place also in a cavity-array system of two or three cavities, where the qubits are placed in different resonators and ultrastrongly interact with them. We observe that this effect can be achieved by probing the system via two different excitation mechanisms: (1) by exciting one of the normal modes of the coupled-cavity array or (2) by selectively exciting only a single cavity. The substantial difference between the two cases is that, while the first case corresponds to the interaction of two qubits with a delocalized field and leads to a deterministic simultaneous excitation process as in Ref.^23^, the second case constitutes one of the simplest examples of a localized system in which the excitation is initially stored in a single resonator. This makes this effect even more counterintuitive, since the excitation is continuously transferred between the nearest-neighbor resonators, while the effect requires both atoms to feel the photon field at the same time in order to take place. In both cases, we study the temporal evolution of the system within both theoretical and numerical approaches, providing a clear and physically intuitive description of the propagation and causality mechanisms underlying this simultaneous excitation phenomena. The process described here could be experimentally realized in state-of-the-art circuit QED systems. Moreover, these effects can find useful applications in quantum information processing and quantum communication protocols, where reliable and controllable entanglement between distant qubits in a quantum network is of fundamental importance^88,89^.

## Models and results

Here we study a quantum system consisting of an array of *N* weakly coupled single-mode resonators, each of them interacting with a two-level atom (e.g., a superconducting qubit). The total Hamiltonian of the system can be written as^23^ (hereafter, $\hbar =1$):

1 $$\begin{aligned} {\hat{H}} = {\hat{H}}_{ q} + {\hat{H}}_{ c} + {\hat{H}}_{\mathrm{cc}}+ {\hat{H}}_{\mathrm{qc}}\, , \end{aligned}$$

where $H_{c}=\sum _{n=1}^{N} \omega _{c}^{(n)} {{\hat{a}}}^\dag _n {\hat{a}}^{}_n$ and $H_{q}=\sum _{n=1}^{N} \omega _{q}^{(n)} {{\hat{\sigma }}}_+^{(n)} {{\hat{\sigma }}}_-^{(n)}$ describe, respectively, the qubit and cavity Hamiltonians in the absence of interaction, ${{\hat{a}}}^\dag _n ({\hat{a}}^{}_n)$ is the bosonic creation (annihilation) operator for the *n*th resonator mode with frequency $\omega _{c}^{(n)}$, and ${{\hat{\sigma }}}_+^{(n)}({{\hat{\sigma }}}_-^{(n)})$ are the raising (lowering) operators for the *n*th qubit with transition frequency $\omega _{q}^{(n)}$. The cavity–cavity interaction Hamiltonian is given by

2 $$\begin{aligned} H_{\mathrm{cc}}= J \sum _{n=1}^{N-1} {\hat{a}}^\dag _{n}{\hat{a}}_{n+1}\, , \end{aligned}$$

where *J* is the next-neighbour hopping rate. We are assuming that the coupling strength *J* between the two cavities is weak, thus we can apply the RWA to the cavity–cavity interaction Hamiltonian ${\hat{H}}_{\mathrm{cc}}$. Finally, the last term of Eq. (1), describing the interaction between the qubits and the cavity modes, reads.

3 $$\begin{aligned} H_{\mathrm{qc}}=\sum _{n=1}^{N} g_{n}\, {\hat{X}}_{n} [\cos (\theta _{n}){{\hat{\sigma }}}_x^{(n)} + \sin (\theta _{n}){{\hat{\sigma }}}_z^{(n)}] \, , \end{aligned}$$

where $g_n\equiv |g_n|e^{i \varphi _n}$ denotes the coupling rate of the *n*th qubit to the corresponding cavity field ${\hat{X}}_{n}\equiv {{\hat{a}}}^\dag _n+ {\hat{a}}^{}_n$, the angles $\theta _{n}$ parametrize the relative contribution of the transverse and longitudinal couplings, while ${{\hat{\sigma }}}_x^{(n)}$ and ${{\hat{\sigma }}}_z^{(n)}$ are the Pauli matrices for the qubits. In circuit QED systems, the angle $\theta _n$ and the transition frequency $\omega _{q}^{(n)}$ can be continuously tuned by changing the magnetic field externally applied to, e.g., a flux qubit (see, e.g., Ref.^43^). An important feature of the interaction Hamiltonian is that it contains terms that do *not* conserve the total number of excitations. Specifically, the *transverse* coupling $\propto {{\hat{\sigma }}}_x$ contains terms like ${\hat{a}}^{}{{\hat{\sigma }}}_-$ and ${{\hat{a}}}^\dag {{\hat{\sigma }}}_+$ which create or annihilate *two* excitation simultaneously; whereas the ${{\hat{\sigma }}}_z$-coupling changes the resonator photon number by one, while leaving the number of qubit excitations unchanged. Notice that the parity of qubit *n* is conserved only for $\theta _n = 0$, corresponding, for a flux qubit, to a zero external flux offset^43^. The terms in the total Hamiltonian which do not conserve the number of excitations in the system can be safely neglected in the weak-coupling regime, where the rotating-wave approximation (RWA) is valid. However, these terms become relevant for systems entering the USC regime, where the coupling strength $g_{n}$ reaches an appreciable fraction of the unperturbed frequencies (here, $\omega _{c}^{(n)}$ and $\omega _{q}^{(n)}$) of the bare systems. One of the most interesting consequences of the presence of these counter-rotating terms is the possibility to coherently couple quantum states with *different* numbers of excitations. These unconventional couplings determine new intriguing physical processes as, for example, multiphoton Rabi oscillations^21^ and the possibility to excite two or more spatially separated atoms with a single photon^23^. Here, we investigate the latter process, considering a fundamentally different setup, consisting of weakly coupled cavity arrays, where each cavity ultrastrongly interacts with a single qubit. Our results show that, owing to the ultrastrong light–matter interaction and the parity-symmetry breaking, it is still possible to simultaneously excite two qubits, even if they are placed in different cavities.

### Two cavities, two qubits and one photon

**Figure 1:**
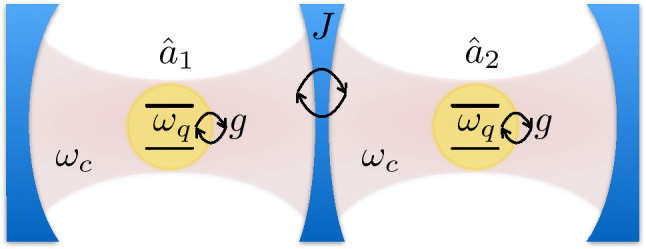
Sketch of the system. Two identical spatially separated optical resonators, with resonance frequency $\omega _{c}$, are weakly coupled together, each one of them ultrastrongly interacting with a single two-level system (qubit) with transition frequency $\omega _{q}$. The photon hopping rate *J* between the two resonators and the light–matter coupling strength *g* are indicated.

We first focus on the study of an array of two identical cavity–qubit systems $\bigl ( \omega _c^{(1)}=\omega _c^{(2)}=\omega _{c}\, ; \,\omega _q^{(1)}=\omega _q^{(2)}=\omega _{q}\, ; \,\theta _{1}=\theta _{2}=\theta \bigr )$, where the two cavities are weakly coupled together and each one of them ultrastrongly interacts with a single qubit (see Fig. 1). In this case, the Hamiltonian of Eq. (1) becomes

4 $$\begin{aligned} {\hat{H}} =\sum _{n=1}^2\left[ \omega _{c} \,{{\hat{a}}}^\dag _n {\hat{a}}^{}_n + \omega _{q}\, {{\hat{\sigma }}}_+^{(n)} {{\hat{\sigma }}}_-^{(n)} +\left| g\right| e^{i \varphi _n} {\hat{X}}_{n} \left( \cos \theta \, {{\hat{\sigma }}}_x^{(n)} + \sin \theta \,{{\hat{\sigma }}}_z^{(n)} \right) \right] + J \left( {\hat{a}}^{}_1 {{\hat{a}}}^\dag _2 + {{\hat{a}}}^\dag _1 {\hat{a}}^{}_2 \right) \,, \end{aligned}$$

The Hamiltonian in Eq. (4) can be conveniently rewritten in terms of the bosonic symmetric and antisymmetric normal modes, also referred to as supermodes defined via the operators ${\hat{a}}^{}_{S(A)}=({\hat{a}}_{1}\pm {\hat{a}}_{2})/\sqrt{2}$, which diagonalise the Hamiltonian

5 $$\begin{aligned} {\hat{H}}_C=\sum \limits _{n=1}^2 \omega _{c} \,{{\hat{a}}}^\dag _n {\hat{a}}^{}_n + J \left( {\hat{a}}^{}_1 {{\hat{a}}}^\dag _2 + {{\hat{a}}}^\dag _1 {\hat{a}}^{}_2 \right) \,, \end{aligned}$$

describing two weakly coupled harmonic oscillators.

In this case, we obtain

6 $$\begin{aligned} \hat{{\mathcal {H}}}= & {} \omega _{A} \,{\hat{a}}^\dag _{A} {\hat{a}}_{A}^{} +\omega _{S} \,{\hat{a}}_{S}^{\dag } {\hat{a}}_{S}^{}+ \omega _{q}\sum _{n=1}^2 {{\hat{\sigma }}}_+^{(n)} {{\hat{\sigma }}}_-^{(n)} \nonumber \\&+ | g | \left[ {\hat{X}}_{A} \left( \cos \theta \, {\hat{\Phi }}_{x}^{-} + \sin \theta \, {\hat{\Phi }}_{z}^{-} \right) +{\hat{X}}_{S} \left( \cos \theta \, {\hat{\Phi }}_{x}^{+} + \sin \theta \, \hat{\Phi }_{z}^{+} \right) \right] \,, \end{aligned}$$

where ${\hat{X}}_{S(A)}\equiv {\hat{a}}^\dag _{S(A)} + {\hat{a}}_{S(A)}^{}$ and ${\hat{\Phi }}_{x(z)}^{\pm }\equiv \left( e^{i \varphi _1}\,{\hat{\sigma }}_{x(z)}^{(1)} \pm e^{i \varphi _2}\, {\hat{\sigma }}_{x(z)}^{(2)}\right) /\sqrt{2}$. The Hamiltonian in Eq. (6) describes two bosonic modes, one symmetric and one antisymmetric with corresponding frequencies $\omega _{S}=\omega _{c}+ J$ and $\omega _{A}=\omega _{c}- J$, both interacting with two qubits. We now diagonalise numerically $\hat{{\mathcal {H}}}$, indicating the resulting energy eigenvalues and eigenstates as $\omega _{i}$ and $|E_i\rangle$, with $i = 0,1, \dots ,$. We label the states such that $\omega _{k}>\omega _{j}$ for $k > j$. In our analysis, we use the notation $|{\mathcal {N}}_A,{\mathcal {N}}_S,q_1,q_2\rangle =|{\mathcal {N}}_A\rangle \bigotimes |{\mathcal {N}}_S\rangle \bigotimes |q_1\rangle \bigotimes |q_2\rangle$ for the eigenstates $|E_i\rangle$, where $q=\left\{ g,e\right\}$ denotes the qubit ground or excited states, respectively, and $|{\mathcal {N}}_{S(A)}\rangle =\left\{ |0\rangle ,|1\rangle ,|2\rangle ,\dots \right\}$ represents the Fock states with a photon occupation number ${\mathcal {N}}$ in the symmetric (antisymmetric) normal mode.

**Figure 2:**
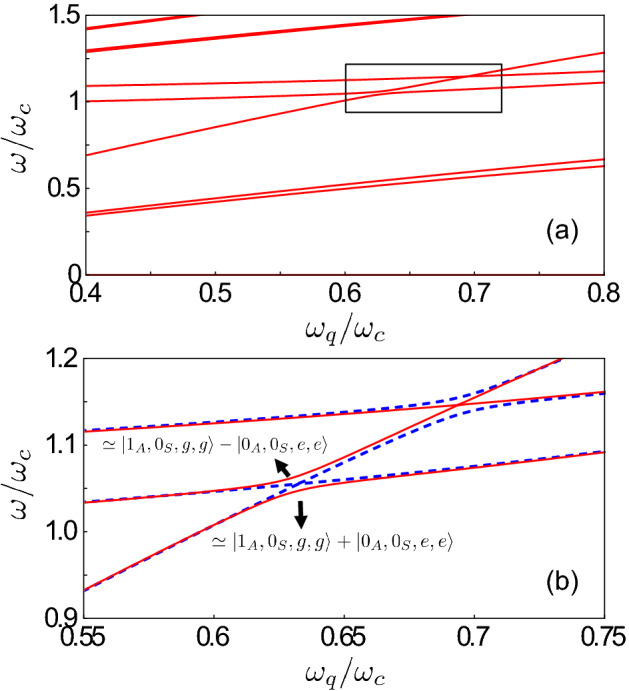
(**a**) Energy differences $\omega _{i0}=\omega _{i}-\omega _{0}$ for the lowest-energy dressed states of $\hat{{\mathcal {H}}}$ as a function of the normalized qubit frequency $\omega _{q}/\omega _{c}$ (which can be experimentally tuned by changing the external flux bias acting on the qubits). We consider a normalized coupling rate $\eta \equiv |g|/\omega _{c}=0.3$ between the qubit and the resonators, while the normalized photon hopping rate between the two resonators is $J/\omega _{c}=0.05$. The phases for the cavity–qubit coupling strengths are set to $\varphi _{1}=0$ and $\varphi _{2}=\pi$, respectively, and the longitudinal interaction coupling term is included by considering a mixing angle $\theta = \pi /6$. (**b**) Enlarged view of the inset in (**a**). When the cavity–qubit coupling strengths have opposite phases ($\varphi _{1}=0,\varphi _{2}=\pi$), the avoided-level crossing (red solid curves) results from the coupling between the states $|1_A,0_S,g,g\rangle$ and $|0_A,0_{S},e,e\rangle$ due to the presence of counter-rotating terms in the system Hamiltonian. The energy splitting reaches its minimum at $\omega _{q} \simeq \omega _{A}/2$. The crossing between the states $|0_A,1_S,g,g\rangle$ and $|0_A,0_{S},e,e\rangle$ at $\omega _{q} \simeq \omega _{S}/2$ indicates that the qubits do not couple with the symmetric normal mode. The complementary result is obtained when the coupling strengths have the same phase (blue dashed curves). In this case, this splitting at $\omega _{q} \simeq \omega _{A}/2$ disappears, while the energy spectrum displays an avoided-level crossing around $\omega _{q} \simeq \omega _{S}/2$, arising from the coherent coupling between the states $|0_A,1_{S},g,g\rangle$ and $|0_A,0_{S},e,e\rangle$.

We set the normalized hopping rate and the light–matter coupling strength as $J/\omega _{\mathrm{c}}=0.05$ and $\eta \equiv |g|/\omega _{\mathrm{c}}=0.3$, respectively. Moreover, we set the phases for the cavity–qubit coupling strengths to $\varphi _{1}=0$ and $\varphi _{2}=\pi$, respectively. This specific phase difference does not affect the dynamics, however it will play an important role for the case of three coupled cavities (see Sect.“”Three cavities, two qubits and one photon). We also consider a mixing angle $\theta = \pi /6$, such that both the longitudinal and transverse contributions to the interaction have comparable values. Figure 2a shows the energy differences $\omega _{i0}=\omega _{i}-\omega _{0}$ for the lowest-energy states as a function of the normalized qubit frequency $\omega _{q}/\omega _{c}$, which can be experimentally tuned by changing the external magnetic flux acting on the qubit. We observe that, when $\omega _{q} \simeq \omega _{A}/2$, the spectrum displays an avoided-level crossing between the states $|E_3\rangle$ and $|E_4\rangle$. It is worth noticing that the resonance condition is quite different from the expected one, i.e., $\omega _{q} = \omega _{A}/2$, because $\omega _q$ and $\omega _A$ are bare resonance frequencies of the matter and light components. The actual physical frequencies are significantly dressed by the interaction (see, e.g.,^21,23,25,79,90^). Note that, just outside this avoided-level crossing region, one level remains flat as a function of the qubit frequency with energy $\omega \approx \omega _{A}$, while the other shows a linear behaviour with $\omega \approx 2 \omega _{q}$. The origin of this splitting is due to the hybridization of the states $|1_A,0_S,g,g\rangle$ and $|0_A,0_{S},e,e\rangle$. When the splitting is at its minimum, these states are well approximated by the superposition states

7 $$\begin{aligned} |E_{3(4)}\rangle =\left( |1_A,0_S,g,g\rangle \pm |0_A,0_{S},e,e\rangle \right) /\sqrt{2}\, . \end{aligned}$$

The (numerically calculated) normalized minimum splitting has a value $2 \Omega _{\mathrm{eff}}/\omega _{c}=16 \times 10^{-3}$, where $\Omega _{\mathrm{eff}}$ is the effective coupling rate between two qubits and one photon. It is important to observe that the coherent coupling between these two states would not be allowed within the RWA, since they have a different number of excitations. Moreover, as the excitation number difference between the two states is odd, parity-symmetry breaking ($\theta \ne 0$) is required in order to observe this splitting. As reported in Ref.^23^, the effective coupling between the states $|E_3\rangle$ and $|E_4\rangle$ can be analytically described by an effective Hamiltonian. Moreover, this coherent coupling is not direct, but can only occur via virtual transitions which are enabled by the counter-rotating terms in $\hat{{\mathcal {H}}}$. In this way, the initial state $|1_A,0_{S},g,g\rangle$ evolves to virtual intermediate states that eventually do not conserve the energy, but it finally evolves to a real energy-conserving state, i.e., $|0_A,0_{S},e,e\rangle$. It is interesting to observe that, for $\omega _{q} \simeq \omega _{S}/2$, the energy spectrum displays a crossing between the levels $|0_A,1_{S},g,g\rangle$ and $|0_A,0_{S},e,e\rangle$, showing that the two qubits do not interact with the symmetric normal mode of the coupled cavities. Indeed, if the coupling strengths $g_{1}$ and $g_{2}$ have opposite signs it can be shown that all the possible intermediate virtual transitions for the process $|0_A,1_{S},g,g\rangle \rightarrow |0_A,0_{S},e,e\rangle$, induced by the interaction term proportional to ${\hat{X}}_{S}$, lead to an intermediate state proportional to $\left| e \rangle \langle e \right| -\left| e \rangle \langle e \right|$, thus giving a vanishing contribution.

Figure 2b shows the comparison of the avoided-level crossing behavior for the cases $\varphi _{1}=0,\varphi _{2}=\pi$ (solid red curves) and $\varphi _{1}=\varphi _{2}=0$ (dashed blue curves). It can be observed that the two choices lead to complementary results. Indeed, when the two coupling strengths have same signs, the splitting at $\omega _{q} \simeq \omega _{A}/2$ disappears, while we observe the presence of an avoided-level crossing around $\omega _{q} \simeq \omega _{S}/2$ arising from the coherent coupling between the states $|0_A,1_{S},g,g\rangle$ and $|0_A,0_{S},e,e\rangle$. This result shows that, depending on the relative signs of the coupling strengths, for $N=2$ the simultaneous excitation of two qubits placed in different resonators can be achieved by coupling the qubits either to the symmetric or antisymmetric normal mode.

In order to fully understand and characterise this process, we fix the qubit frequency at the value where the splitting, as shown in Fig. 2a, between the energy levels corresponding to the eigenstates $|E_{3}\rangle$ and $|E_{4}\rangle$ is minimum and consider the system initially prepared in the one-photon state $|1_A\rangle \equiv |1_A,0_{S},g,g\rangle$. As we will see later, the preparation of this state can be experimentally achieved by sending an appropriate electromagnetic Gaussian pulse to the first cavity.

**Figure 3:**
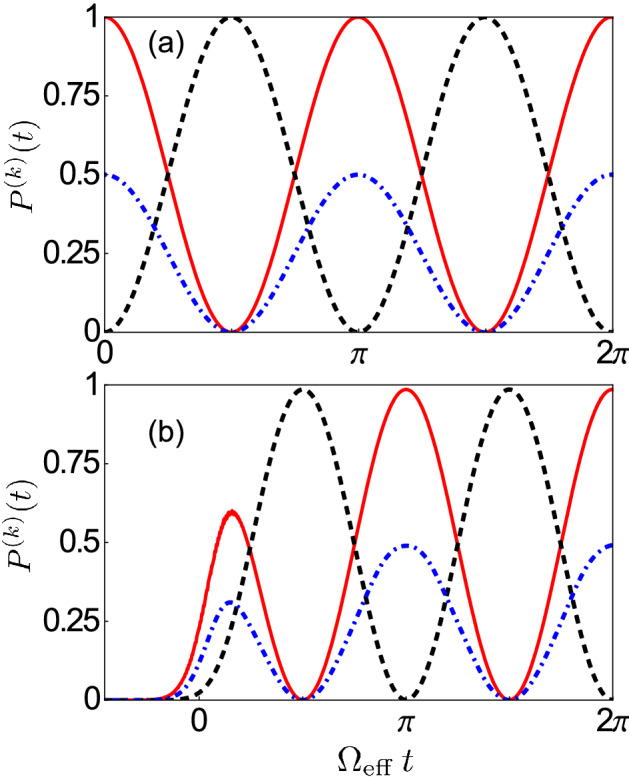
(**a**) Time evolution of the occupation probabilities $P^{(k)}(t)\equiv \langle \hat{{\mathcal {P}}}_{k} \rangle$, with $\hat{{\mathcal {P}}}_{k}= \left| k \rangle \langle k \right|$, for the single-photon states $|1_A\rangle$ (red solid curve) and $|1_1\rangle$ (blue dot-dashed curve), together with the probability ${\mathcal {P}}^{(ee)} (t)$ of having both qubits simultaneously excited (black dashed curve) for the system initially prepared in the state $|1_A\rangle \equiv |1_A,0_{S},g,g\rangle$. Vacuum Rabi oscillations showing a reversible excitation exchange process between the qubits and the resonators are clearly visible. The joint absorption of an antisymmetric cavity photon by the two qubits is achieved after a Rabi half period $\Omega _{\mathrm{eff}}\,t=\pi /2$, with the excitation probability ${\mathcal {P}}^{(ee)}$ approaching one, even if they are placed in different resonators. Here, the effects of dissipation have not been included. (**b**) Temporal evolution of the same occupation probabilities $P^{(k)}(t)$ considered in (**a**), but after the arrival of a narrow Gaussian pulse exciting the first cavity when the system is initially prepared in its ground state. The amplitude and the central frequency of the pulse are $A/\omega _c = 4.2\times 10^{-2}$ and $\omega _d=(\omega _{30}+\omega _{40})/2$, respectively.

Figure 3a displays the numerically calculated time evolution of the occupation probabilities $P^{(k)}(t)\equiv \langle \hat{{\mathcal {P}}}_{k} \rangle$, with $\hat{{\mathcal {P}}}_{k}= \left| k \rangle \langle k \right|$, for the one-photon states $|1_A\rangle$ (i.e., one photon in the antisymmetric normal mode) and $|1_1\rangle$ (one photon in the first cavity), together with the probability ${\mathcal {P}}^{(ee)} (t)$ of having both qubits simultaneously excited. In terms of the dressed energy eigenstates of the system, these states can be expressed, respectively, as

8 $$\begin{aligned} |1_A\rangle&=\left( |E_3\rangle +|E_4\rangle \right) /\sqrt{2}\, , \end{aligned}$$

9 $$\begin{aligned} |1_1\rangle&=\left( |E_3\rangle +|E_4\rangle +\sqrt{2} \,|E_5\rangle \right) /2\, , \end{aligned}$$

10 $$\begin{aligned} |e,e\rangle&=\left( |E_3\rangle -|E_4\rangle \right) /\sqrt{2}\,, \end{aligned}$$

where for concision, we omitted in the last equation the photonic states, which are intended to be in the ground state. Here, $|e,e \rangle$ stands for $|0_A, 0_S,e,e \rangle$. As expected, since $|1_A\rangle =\left( |1_1,0_2\rangle -|0_1,1_2\rangle \right) |g,g \rangle /\sqrt{2}$, at the initial instant of time ${\mathcal {P}}^{( 1_{ A})} (0)=1$, while ${\mathcal {P}}^{( 1_1)} (0)=1/2$. As time evolves, vacuum Rabi oscillations, showing the reversible excitation exchange process between the qubits and the resonators, are clearly visible. Specifically, we observe that, after a Rabi half period $\Omega _{\mathrm{eff}}\,t=\pi /2$, one photon in the antisymmetric cavity mode is jointly absorbed by the two qubits, even if they are placed in different resonators. Moreover, the excitation probability ${\mathcal {P}}^{(ee)}$ approaches one, showing that the multiatom absorption of a single photon can essentially be deterministic. Notice that, in order to provide a clearer description of this counter-intuitive excitation mechanism, the effects of dissipation have not been taken into account. This approximation becomes experimentally reasonable when the system loss rates are smaller than the frequency splitting between the levels involved at the avoided-level crossing, so that the first Rabi cycles are almost not affected by dissipation. A similar oscillating dynamics can be obtained for the system initially prepared in the state $| e,e \rangle$. In this case, the two qubits will jointly and coherently release their energy to the cavity. The time evolution of the system will be as shown in Fig. 3a, but with the initial time $t= \pi / (2\Omega _{\mathrm{eff}})$.

As mentioned before, instead of starting from the ideal initial state $|1_A\rangle$, we now consider a more realistic case where the system is initially in its ground state $|E_0\rangle =|0_A,0_S,g,g\rangle$ and study a direct excitation of the first cavity by an electromagnetic Gaussian pulse. The corresponding driving Hamiltonian is

11 $$\begin{aligned} {\hat{H}}_{d}={\mathcal {E}}(t)\cos (\omega _d \,t){\hat{X}}_{1} \, , \end{aligned}$$

where $${\hat{X}}_1= {\hat{X}}_{S}+{\hat{X}}_{A}$$ and

12 $$\begin{aligned} {\mathcal {E}}(t)=A \exp \left[ - (t-t_0)^2/(2 \tau ^2) \right] / (\tau \sqrt{2} \pi )\,, \end{aligned}$$

with *A* and $\tau$ the amplitude and the standard deviation of the Gaussian pulse, respectively. The central frequency of the pulse has been chosen to be in the middle of the two split transition energies $\omega _d=(\omega _{30}+\omega _{40})/2$. The pulse bandwidth must be sufficiently narrow in order to ensure that only the states $|E_{3}\rangle$ and $|E_{4}\rangle$ are excited, so that the pulse can directly excite the state $|1_A\rangle =( |E_3\rangle +|E_4\rangle ) /\sqrt{2}$, and the symmetric mode $|1_S \rangle$ is not excited. This corresponds to a pulse duration $\sim \tau$ significantly larger than the transfer time $\sim \pi /(2J)$ of photons from one cavity to the other. In this way, even if the system is fed through a single cavity only, both cavities are actually excited simultaneously without any causality issue, because the excitation time $\tau$ is larger than the transfer time $\sim \pi /(2 J)$. With this excitation scheme, the resulting dynamics is very similar to the case of two atoms in a single cavity^23^.

Figure 3b shows the dynamics of the occupation probabilities ${\mathcal {P}}^{( 1_{ A})}$, ${\mathcal {P}}^{( 1_{ 1})}$, and ${\mathcal {P}}^{( ee)}$ after the arrival of the $\pi$-like Gaussian pulse initially exciting the first cavity described by the Hamiltonian in Eq. (11). We observe that, since the pulse time width is not much narrower than the Rabi period, after the arrival of the pulse, the antisymmetric normal mode is not completely populated and the excitation is partially transferred to the qubits. Therefore, the first peak of the antisymmetric normal mode occupation probability in Fig. 3b is slightly lower than the second one. Once the antisymmetric mode is completely populated, the dynamics of vacuum Rabi oscillations, showing the reversible excitation exchange between one photon in the antisymmetric normal mode and the two qubits, is the same as in Fig. 3a. It is also worth noticing that, in the absence of any system nonlinearity as, e.g., in the case of a system empty (without atoms) resonators, coherent excitation, as described by the Hamiltonian in Eq. (11), would give rise to a coherent intra-cavity field, not to a single-photon Fock state. However, the very strong interaction of the cavity array with the two atoms induces an anharmonicity to the level structure which is able to prevent the resonant excitation of higher-photon states (photon-blockade, see, e.g.,^91,92^). In the case of lower atom-cavity coupling strengths, the photonic system could be complemented by additional Kerr nonlinearities, which are able to induce photon blockade^23^.

**Figure 4:**
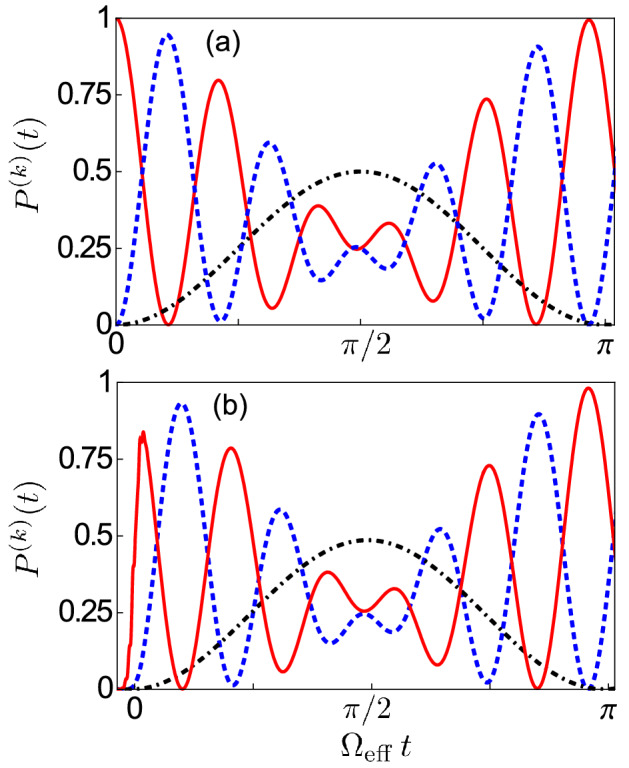
(**a**) Time evolution of the occupation probabilities ${\mathcal {P}}^{( 1_1)} (t)$ (red solid curve), ${\mathcal {P}}^{( 1_2)} (t)$ (blue dotted curve), and ${\mathcal {P}}^{( ee)} (t)$ (black dot-dashed curve) with the system initially prepared in the one-photon state $|\psi _1\rangle$ given in Eq. (13). As the time evolves, the expected photon hopping between the two weakly-coupled cavities is accompanied by the simultaneous excitation of the two qubits by a single-cavity photon. The maximum probability ${\mathcal {P}}^{( ee)}=1/2$ is achieved at $t=\pi /(2\Omega _{\mathrm{eff}})$. The corresponding values ${\mathcal {P}}^{( 1_1)}={\mathcal {P}}^{( 1_2)}=1/4$ for the cavity occupation probabilities can be explained considering that at $t=\pi /(2\Omega _{\mathrm{eff}})$ the system is in the state $|\psi _2\rangle$ given in Eq. (15). Here, the effects of losses have not been taken into account. (**b**) Temporal evolution of the same occupation probabilities $P^{(k)}(t)$ considered in (**a**), after the arrival of a broad Gaussian pulse exciting the first cavity when the system is initially prepared in its ground state. The amplitude and the central frequency of the pulse are $A/\omega _c = 0.27$ and $\omega _d\simeq ({\bar{\omega }}_{34}+\omega _{50})/2$, with ${\bar{\omega }}_{34}=(\omega _{30} + \omega _{40})/2$.

The simultaneous excitation process of the two qubits with a single photon can also be achieved by initially exciting only the first cavity. This can be realized experimentally by feeding the cavity with a fast Gaussian pulse (with respect to the transfer time between the cavities). Specifically, in order to completely populate the first cavity before the excitation is transferred to the second one, the pulse bandwidth $\Gamma = \pi /\tau$ has to be much larger than the energy splitting $\Omega =2J$ between the two cavity normal modes, i.e., $\Gamma \gg \Omega$. Figure 4a shows the time evolution of the occupation probabilities ${\mathcal {P}}^{( 1_1)} (t)$, ${\mathcal {P}}^{( 1_2)} (t)$, and ${\mathcal {P}}^{( ee)} (t)$ with the system initially prepared in the state

13 $$\begin{aligned} |\psi _1\rangle =\left( |1_A,0_{S},g,g\rangle +|0_A,1_{S},g,g\rangle \right) /\sqrt{2}=|1_1\rangle . \end{aligned}$$

Besides the expected photon hopping between the two weakly-coupled cavities, as the time evolves, we observe that this excitation-transfer process is accompanied by the simultaneous excitation of the two qubits. However, in contrast to the previous case, where the probability for the qubits to be simultaneously excited reached one, here we observe the maximum probability ${\mathcal {P}}^{( ee)}=1/2$ at $t=\pi /(2\Omega _{\mathrm{eff}})$. This difference can be explained by considering that a direct excitation of the first cavity only corresponds to an equal weight superposition of both the symmetric and antisymmetric normal modes (see Eq. 13), each of them carrying half of the total initial excitation. However, since the qubits do not interact with the symmetric mode, only the antisymmetric excitation contribution can be transferred to the two qubits, resulting into a maximum joint qubit excitation probability ${\mathcal {P}}^{( ee)}=1/2$. This result indicates that the simultaneous excitation of two qubits with one photon is still possible, but the qubits cannot be excited with probability ${\mathcal {P}}^{( ee)}=1$. Moreover, the corresponding values ${\mathcal {P}}^{( 1_1)}={\mathcal {P}}^{( 1_2)}=1/4$ for the cavity occupation probabilities can be explained by directly following the dynamics of the system. Indeed we observe that, when the system is prepared in the superposition state $|\psi _1\rangle$, then the state $|0_A,1_{S},g,g\rangle$ evolves freely, as an eigenstate of $\hat{{\mathcal {H}}}$. On the contrary, since the qubits are coupled with the antisymmetric normal mode, once again we observe the coherent energy exchange process $|1_A,0_{S},g,g\rangle \leftrightarrow |0_A,0_{S},e,e\rangle$ so that at $t=\pi /(2\Omega _{\mathrm{eff}})$ the system is in the state

14 $$\begin{aligned} |\psi _2\rangle =\left( |0_A,0_{S},e,e\rangle +|0_A,1_{S},g,g\rangle \right) /\sqrt{2}. \end{aligned}$$

In terms of the energy eigenstates of the Hamiltonian of the uncoupled system ($J=g=0$), the state $|\psi _2\rangle$ can be expressed as

15 $$\begin{aligned} |\psi _2\rangle = \frac{1}{\sqrt{2}} |0_1,0_2,e,e\rangle + \frac{1}{2} \left( |1_1,0_2,g,g\rangle +|0_1,1_2,g,g\rangle \right) , \end{aligned}$$

thus explaining the observed values, in Fig. 4a, for the occupation probabilities ${\mathcal {P}}^{( 1_1)},{\mathcal {P}}^{( 1_2)}$ and ${\mathcal {P}}^{( ee)}$.

Finally, even for this case we consider the system initially prepared in its ground state and study the system dynamics after the arrival of a Gaussian pulse exciting the first cavity and described by Eq. (11). Unlike the previous case, in order to excite the states $|E_3\rangle$, $|E_4\rangle$ and $|E_5\rangle$ we need to apply a broad-bandwidth Gaussian pulse with central frequency $\omega _d\simeq ({\bar{\omega }}_{34} + \omega _{50})/2$, where ${\bar{\omega }}_{34}=(\omega _{30} + \omega _{40})/2$. The temporal evolution of the occupation probabilities, after the arrival of the Gaussian pulse feeding the first cavity, is shown in Fig. 4b. It is interesting to observe that, in the absence of the qubits, the excitation would simply be transferred from one cavity to the another. Indeed, the simultaneous excitation process can take place because, except for some specific instants of time, in which the excitation is totally localized in one cavity only, the field is delocalized over the two adjacent cavities. For this reason, both qubits feel the electric field simultaneously reaching the maximum excitation probability when the field is equally distributed between the two cavities, even if they are detuned from the cavity mode.

The causal mechanisms underlying the possibility for a single photon to be jointly absorbed by the two qubits under different excitation processes can be explained by considering a simpler system constituted by the two weakly coupled cavities only. If the system is initially prepared in the state $|1_1,0_2\rangle$, the complete population transfer $|1_1,0_2\rangle \rightarrow |0_1,1_2\rangle$ will take place after a period $T=\pi / (2J)$. From an experimental point of view, if we consider the system to be prepared in its ground state $|0_1,0_2\rangle$, the direct excitation of the antisymmetric normal mode $|-\rangle =\left( |1_1,0_2\rangle -|0_1,1_2\rangle \right) /\sqrt{2}$ can be realized by feeding the first cavity with a Gaussian pulse whose bandwidth $\Gamma$ has to be smaller than the energy splitting between the two normal modes, *i.e.*, $\Gamma \ll 2J$. Since in this case for the temporal pulse width $\delta t \gg T$, the photon can travel back and forth between the two cavities before the normal mode becomes fully populated and the electric field always results delocalized over the whole cavity array.

In contrast to this, the realization of a localized cavity mode (e.g., the state $|1_1,0_2\rangle$) can be achieved by feeding the first cavity with a fast optical Gaussian pulse whose bandwidth has to be large enough to excite the superposition state $\left( |+\rangle +|-\rangle \right) /\sqrt{2}$. The condition $\Gamma \gg 2J$ ensures that, being $\delta t \ll T$, the pulse duration is short enough to localise the electric field in the first cavity. Then, due to the cavity–cavity interaction the excitation-transfer process will take place and the electric field will be completely delocalized over the two cavities only at the instants of time when ${\mathcal {P}}^{( 1_1)}={\mathcal {P}}^{( 1_2)}=1/2$.

### Three cavities, two qubits and one photon

**Figure 5:**
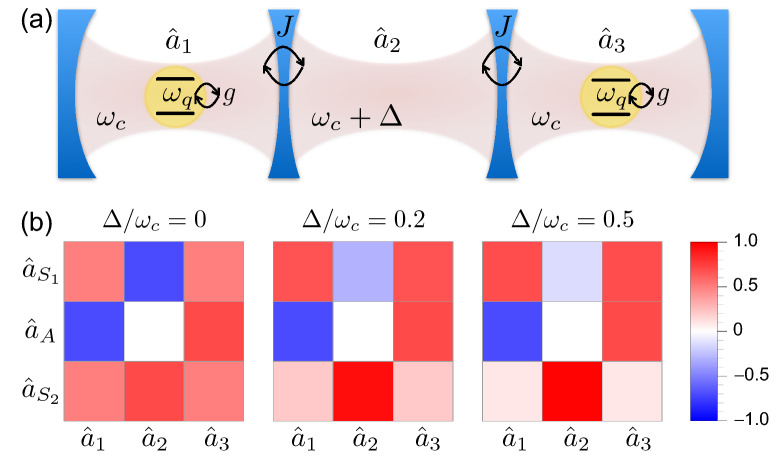
(**a**) Sketch of an array of three optical resonators weakly coupled with their nearest neighbours. The two resonant end cavities ultrastrongly interact with a single qubit with transition frequency $\omega _{q}$, while the central cavity is empty and can be detuned by an amount $\Delta$. The photon hopping rate between the three resonators and the light–matter coupling strength are indicated with *J* and *g*, respectively. (**b**) Density plot of the transformation matrix diagonalizing ${\hat{H}}_{C}^{\prime }$, evaluated for different values of the normalized detuning $\Delta /\omega _{c}$ for $J/\omega _{c}=0.05$ and $\eta \equiv |g|/\omega _{c}=0.3$. While the antisymmetric normal mode is not affected by the detuning, the spatial profile of the two symmetric normal modes changes significantly with increasing values of $\Delta$. Specifically, for higher values of the detuning, the lower-energy mode ${\hat{a}}_{\text {S}_1}$ becomes totally delocalized in the end cavities, while the higher-energy mode ${\hat{a}}_{\text {S}_2}$ is completely localized in the central cavity.

Here we extend the previous analysis to a more complex system consisting of an array of three weakly-coupled cavities, where the two end cavities ultrastrongly interact with a single qubit while the central cavity is empty (as shown in Fig. 5a). The Hamiltonian describing the system is

16 $$\begin{aligned} {\hat{H}}= & {} \sum _{n=1}^3 \omega _{c}^{(n)} \,{{\hat{a}}}^\dag _n {\hat{a}}^{}_n + \sum _{n=1}^2\biggl [\omega _{q}\, {{\hat{\sigma }}}_+^{(n)} {{\hat{\sigma }}}_-^{(n)} + J \left( {{\hat{a}}}^\dag _{n} {\hat{a}}^{}_{n+1} + {{\hat{a}}}^\dag _{n+1} {\hat{a}}^{}_{n} \right) \nonumber \\&+\left| g\right| e^{i \varphi _{(2n-1)}} {\hat{X}}_{(2n-1)} \left( \cos \theta \, {{\hat{\sigma }}}_x^{(n)} + \sin \theta \,{{\hat{\sigma }}}_z^{(n)} \right) \biggr ], \end{aligned}$$

where the normalized hopping rate and light–matter coupling strength are, respectively, $J/\omega _{\mathrm{c}}=0.05$ and $\eta \equiv |g|/\omega _{\mathrm{c}}=0.3$, and the presence of the longitudinal interaction term is taken into account by considering a mixing angle $\theta =\pi /6$. Due to the small value of the normalized hopping rate $J/\omega _{c}$, we apply the RWA to the cavity–cavity interaction term. Moreover, we consider the case in which the two end cavities are resonant $(\omega _{c}^{(1)}=\omega _{c}^{(3)}=\omega _{c})$, while the central cavity can be detuned by an amount $\Delta$. The interaction between these three cavities is described by the Hamiltonian

17 $$\begin{aligned} {\hat{H}}_C^{\prime }= \omega _{c} \,({{\hat{a}}}^\dag _1 {\hat{a}}^{}_1 + {{\hat{a}}}^\dag _3 {\hat{a}}^{}_3) +(\omega _{c}+\Delta ) \,{{\hat{a}}}^\dag _2 {\hat{a}}^{}_2+ J \sum \limits _{n=1}^2 ( {{\hat{a}}}^\dag _{n} {\hat{a}}^{}_{n+1} + {{\hat{a}}}^\dag _{n+1} {\hat{a}}^{}_{n}) , \end{aligned}$$

which produces three normal modes: an antisymmetric flat state, and two symmetric modes whose energy splitting is $\Omega =\sqrt{8J^2+\Delta ^2}$. The transformation, which diagonalizes ${\hat{H}}_{C}^{\prime }$, can be written in matrix form:

18 $$\begin{aligned} \begin{pmatrix} {\hat{a}}_{\text {S}_1}\\ {\hat{a}}_{A}\\ {\hat{a}}_{\text {S}_2} \end{pmatrix}=\begin{pmatrix} 2J/{\mathcal {N}}_{-} &{} \bigl (\Delta -\Omega \bigr ) / {\mathcal {N}}_{-} &{}2J/{\mathcal {N}}_{-} \\ -1/\sqrt{2} &{} 0 &{} 1/\sqrt{2} \\ 2J/{\mathcal {N}}_{+} &{} \bigl (\Delta +\Omega \bigr ) / {\mathcal {N}}_{+} &{} 2J/{\mathcal {N}}_{+} \end{pmatrix} \begin{pmatrix} {\hat{a}}_{1}\\ {\hat{a}}_{2}\\ {\hat{a}}_{3} \end{pmatrix}\, , \end{aligned}$$

with ${\mathcal {N}}_{\pm } = \left[ 8J^2+(\Delta \pm \Omega )^2 \right] ^{1/2}$. In Eq. (18), ${\hat{a}}_{A}$ and ${\hat{a}}_{\text {S}_1(\text {S}_2)}$ are, respectively, the bosonic annihilation operators for the antisymmetric state and the two symmetric normal modes whose corresponding frequencies are $\omega _A = \omega _c$ and $\omega _{\text {S}_{1,2}}=\left( 2 \omega _{{\mathrm{c}}}+\Delta \mp \Omega \right) /2$.

The effect of the detuning on the spatial profile of the three normal modes is displayed in Fig. 5b. We observe that, unlike the antisymmetric mode, which is not affected by the detuning, the *spatial* profile of the two symmetric normal modes changes significantly with increasing values of $\Delta$, and also exhibits an opposite behavior. Specifically, while the lower-energy mode ${\hat{a}}_{\text {S}_1}$ becomes totally delocalized in the end cavities, the higher-energy mode ${\hat{a}}_{\text {S}_2}$ completely localizes in the central cavity. This preliminary analysis suggests that the choice of coupling the two qubits to the antisymmetric mode, which is both detuning-independent and delocalized in the two end cavities where the qubits are placed, is the best strategy for achieving the desired effect of simultaneous excitation of two qubits with a single photon. Focusing on the resonant case $\Delta =0$, the Hamiltonian of Eq. (16) can be conveniently rewritten in terms of the normal mode operators as

19 $$\begin{aligned} \hat{{\mathcal {H}}}^{\prime }= & {} \sum _{k\in [\text {S}_1,\text {S}_2,A]}^{} \omega _{k} \,{\hat{a}}_{k}^{\dag } {\hat{a}}_{k}^{}+\, \omega _{q}\sum _{n=1}^2 {{\hat{\sigma }}}_+^{(n)} {{\hat{\sigma }}}_-^{(n)}\nonumber \\&+ |g|\left[ \frac{1}{\sqrt{2}}\left( {\hat{X}}_{\text {S}_2} - {\hat{X}}_{\text {S}_1}\right) \left( \cos \theta \, {\hat{\Phi }}_{x}^{+} + \sin \theta \, {\hat{\Phi }}_{z}^{+} \right) - {\hat{X}}_{A} \left( \cos \theta \, {\hat{\Phi }}_{x}^{-} + \sin \theta \, \hat{\Phi }_{z}^{-} \right) \right] , \end{aligned}$$

where $${\hat{X}}_{A}\equiv {\hat{a}}^\dag _{A} + {\hat{a}}_{A}^{}$$, $${\hat{X}}_{\text {S}_1(\text {S}_2)}\equiv {\hat{a}}^\dag _{\text {S}_1(\text {S}_2)} + {\hat{a}}_{\text {S}_1(\text {S}_2)}^{}$$, and $$\hat{\Phi }_{x(z)}^{\pm }\equiv \left( e^{i \varphi _1}\,{\hat{\sigma }}_{x(z)}^{(1)} \pm e^{i \varphi _3}\, {\hat{\sigma }}_{x(z)}^{(2)}\right) /\sqrt{2}$$.

**Figure 6:**
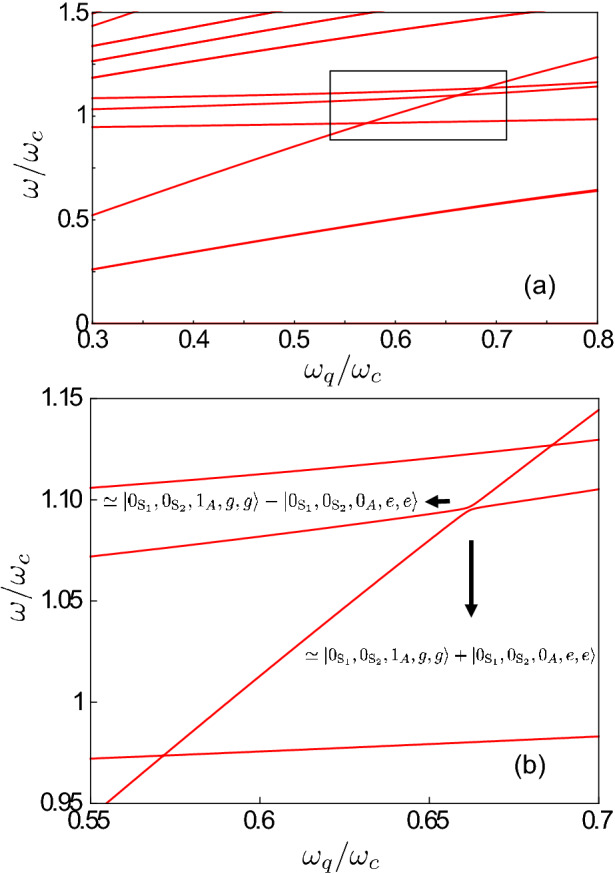
Energy differences $\omega _{i0}=\omega _{i}-\omega _{0}$ for the lowest-energy dressed states of ${\mathcal {H}}^{\prime }$ as a function of the normalized qubit frequency $\omega _{q}/\omega _{c}$ in the absence of detuning ($\Delta =0$). We set the normalized qubit-resonator coupling rate and the inter-cavity photon hopping rate to $\eta \equiv |g|/\omega _{c}=0.3$ and $J/\omega _{c}=0.05$, respectively. The phases for the cavity–qubit coupling strengths are $\varphi _{1}=0$ and $\varphi _{3}=\pi$, while the longitudinal interaction coupling term is included by considering a mixing angle $\theta = \pi /6$. (**b**) Enlarged view of the inset in (**a**). When the cavity–qubit coupling strengths are opposite in phase ($\varphi _{1}=0,\varphi _{3}=\pi$), the avoided level crossing results from the coupling between the states $|0_{\text {S}_1},0_{\text {S}_2},1_{A},g,g\rangle$ and $|0_{\text {S}_1},0_{\text {S}_2},0_{A},e,e\rangle$, due to the presence of counter-rotating terms in the system Hamiltonian. The energy splitting reaches its minimum at $\omega _{q} \simeq \omega _{A}/2$. The presence of two energy-level crossings corresponding to the qubit frequencies $\omega _{q} \simeq \omega _{\text {S}_1}/2$ and $\omega _{q} \simeq \omega _{\text {S}_2}/2$, both involving the state $|0_{\text {S}_1},0_{\text {S}_2},0_{A},e,e\rangle$, indicates that the two qubits do not couple with the two symmetric normal modes.

Figure 6a shows the energy differences $\omega _{i0}=\omega _{i}-\omega _{0}$ for the lowest-energy eigenstates $|E_i\rangle$ (with $i = 0,1, \dots$) of $\hat{{\mathcal {H}}}^{\prime }$, numerically calculated as a function of the normalized qubit frequency $\omega _{q}/\omega _{c}$ by setting the phases of the two cavity–qubit coupling strengths to $\varphi _{1}=0$ and $\varphi _{3}=\pi$, respectively. Here we use the notation $|{\mathcal {N}}_{\text {S}_1},{\mathcal {N}}_{\text {S}_2},{\mathcal {N}}_A,q_1,q_2\rangle =|{\mathcal {N}}_{\text {S}_1}\rangle \bigotimes |{\mathcal {N}}_{\text {S}_2}\rangle \bigotimes |{\mathcal {N}}_A\rangle \bigotimes |q_1\rangle \bigotimes |q_2\rangle$ for the eigenstates $|E_i\rangle$, where $q=\left\{ g,e\right\}$ denote the qubit ground or excited states, respectively, and $|{\mathcal {N}}_{k}\rangle =\left\{ |0\rangle ,|1\rangle ,|2\rangle ,\dots \right\}$, with $k\in [\text {S}_1,\text {S}_2,A]$, represents the Fock state with photon occupation ${\mathcal {N}}_{k}$ in the corresponding normal mode.

Note that the energy spectrum presents a more complicated structure with respect to the case of the two cavity–qubit array. Specifically, two energy-level crossings, both involving the state $|0_{\text {S}_1},0_{\text {S}_2},0_{A},e,e\rangle$ (which displays a linear behavior with $\omega \approx 2 \omega _{q}$), can be observed in correspondence to the qubit frequencies $\omega _{q} \simeq \omega _{\text {S}_1}/2$ and $\omega _{q} \simeq \omega _{\text {S}_2}/2$. The other states involved in the two energy-level crossings are, respectively, $|1_{\text {S}_1},0_{\text {S}_2},0_{A},g,g\rangle$ and $|0_{\text {S}_1},1_{\text {S}_2},0_{A},g,g\rangle$, indicating that when the cavity–qubit coupling strengths have opposite phases, the qubits do not couple with the two symmetric normal modes. Interestingly, in the region between these two-level crossings, an apparent additional one between the levels $|E_{4}\rangle$ and $|E_{5}\rangle$ appears at $\omega _{q} \simeq \omega _{A}/2$. Actually, what appears as a crossing on this scale turns out to be an avoided-level crossing on an enlarged view, as in Fig. 6b. This splitting, which has a normalized value $2 \Omega _{{\mathrm{eff }}}/\omega _{\mathrm{c}}=2\times 10^{-3}$ at its minimum, clearly originates from the hybridization of the states $|0_{\text {S}_1},0_{\text {S}_2},0_{A},e,e\rangle$ and $|0_{\text {S}_1},0_{\text {S}_2},1_{A},g,g\rangle$. The resulting states are well approximated by

20 $$\begin{aligned} |E_{4(5)}\rangle =(|0_{\text {S}_1},0_{\text {S}_2},1_{A},g,g\rangle \pm |0_{\text {S}_1},0_{\text {S}_2},0_{A},e,e\rangle )/\sqrt{2}. \end{aligned}$$

It is important to observe that, similarly to the two cavity–qubit array case, the coherent coupling between these two states would neither be allowed within the RWA, nor in the absence of the longitudinal interaction term $(\theta =0)$. Moreover, the states $|0_{\text {S}_1},0_{\text {S}_2},0_{A},e,e\rangle$ and $|0_{\text {S}_1},0_{\text {S}_2},1_{A},g,g\rangle$ do not couple directly, but the process occurs via intermediate energy non-conserving processes enabled by the counter-rotating terms in $\hat{{\mathcal {H}}}^{\prime }$. It is interesting to observe that, when the coupling strengths are opposite in phase, all the possible intermediate virtual transitions for the process $|0_{\text {S}_1},0_{\text {S}_2},1_{A},g,g\rangle \rightarrow |0_{\text {S}_1},0_{\text {S}_2},0_{A},e,e\rangle$ are induced by the interaction term proportional to ${\hat{X}}_{A}$, while the term proportional to $\left( {\hat{X}}_{\text {S}_2} - {\hat{X}}_{\text {S}_1}\right)$ gives vanishing contributions. The choice of same coupling strength phases $\left( \varphi _1 = \varphi _3\right)$ would lead to the complementary situation with the two qubits decoupled from the antisymmetric mode and simultaneously interacting with the two symmetric modes ${\hat{a}}_{\text {S}_1}$ and ${\hat{a}}_{\text {S}_2}$. In this case, the simultaneous excitation of the two qubits with a single photon can occur via two different processes ($|1_{\text {S}_1},0_{\text {S}_2},0_{A},g,g\rangle \rightarrow |0_{\text {S}_1},0_{\text {S}_2},0_{A},e,e\rangle$ and $|0_{\text {S}_1},1_{\text {S}_2},0_{A},g,g\rangle \rightarrow |0_{\text {S}_1},0_{\text {S}_2},0_{A},e,e\rangle$). However, since the symmetric normal modes are mainly localized in the central empty cavity, the energy splittings of the avoided-level crossings between these states, as well as the corresponding effective couplings, are much smaller.

#### Dynamics after exciting the antisymmetric mode

In order to fully understand the excitation transfer between a single photon and two qubits in a three cavity–qubit array, we now study the dynamics of the system initially prepared in the one-photon state $|1_A\rangle \equiv |0_{\text {S}_1},0_{\text {S}_2},1_{A},g,g\rangle$, fixing the qubit frequency at the value where the splitting in Fig. 6b between levels $|E_{4}\rangle$ and $|E_{5}\rangle$ is minimum. Moreover, for the sake of simplicity, we consider the system loss rates to be significantly smaller than the frequency splitting between the levels involved in the avoided-level crossing, so that the effect of dissipation can be neglected.

**Figure 7:**
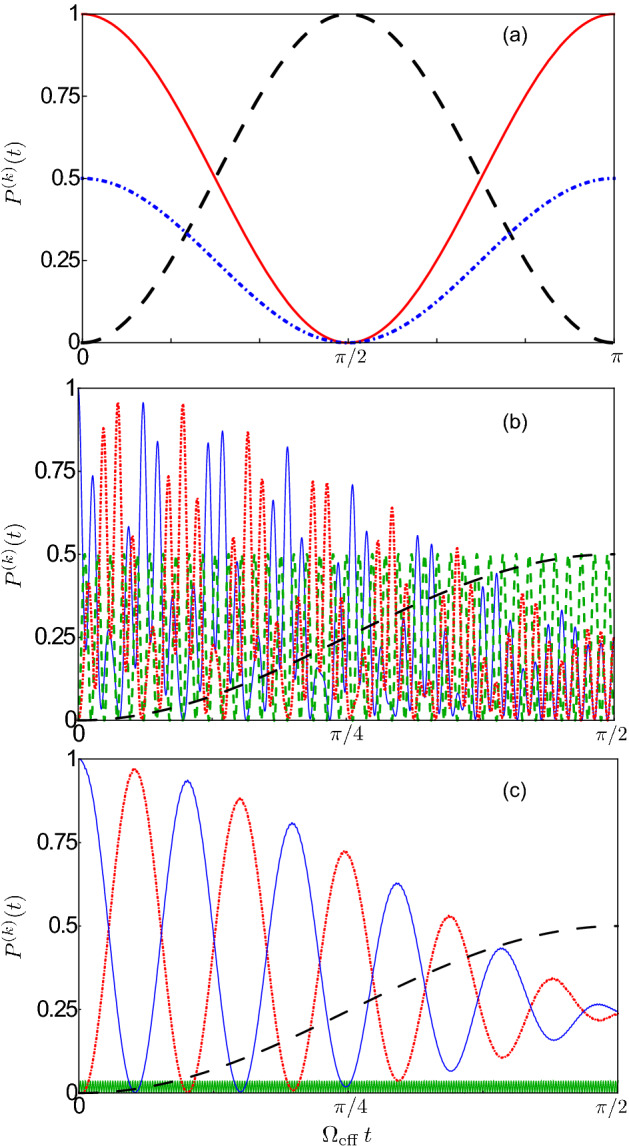
(**a**) Time evolution of the occupation probabilities $P^{(k)}(t)\equiv \langle \hat{{\mathcal {P}}}_{k} \rangle$, with $\hat{{\mathcal {P}}}_{k}= \left| k \rangle \langle k \right|$, for the one-photon states $|1_A\rangle$ (red solid curve) and $|1_1\rangle$ (blue dot-dashed curve), together with the probability ${\mathcal {P}}^{(ee)} (t)$ of having both qubits simultaneously excited (black dashed curve). Here, the system is initially prepared in the one-photon state $|1_A\rangle \equiv |0_{\text {S}_1},0_{\text {S}_2},1_{A},g,g\rangle$ and in the absence of detuning ($\Delta =0$). As the times evolves, the excitation is progressively transferred to the two qubits at the same time, until the maximum simultaneous qubit excitation (${\mathcal {P}}^{( ee)}=1$) by a single photon is reached at $t=\pi /(2 \Omega _{\mathrm{eff}})$. (**b**) Temporal dynamics of the occupation probabilities $P^{(1_1)}$ (blue solid curve), $P^{(1_2)}$ (green dashed curve) and $P^{(1_3)}$ (red dot-dashed curve), together with the probability ${\mathcal {P}}^{(ee)}$ (black dashed curve) of having both qubits simultaneously excited when the system is initially prepared in the one-photon state $|\psi _1\rangle =|1_1\rangle$ of the first resonator with $\Delta =0$. (**c**) Temporal dynamics of the same occupation probabilities $P^{(k)}(t)$ considered in (**b**) but in the presence of the detuning $\Delta /\omega _{c}=0.5$.

The numerically calculated time evolution of the occupation probabilities $P^{(k)}(t)\equiv \langle \hat{{\mathcal {P}}}_{k} \rangle$, with $\hat{{\mathcal {P}}}_{k}= \left| k \rangle \langle k \right|$, for the one-photon states $|1_A\rangle$ (a single photon in the antisymmetric normal mode), $|1_1\rangle$ and $|1_3\rangle$ (a single photon in the first and third cavities, respectively), together with the probability ${\mathcal {P}}^{(ee)} (t)$ of having both qubits simultaneously excited, are displayed in Fig. 7a. As expected, ${\mathcal {P}}^{( 1_{ A})} (0)=1$ at the initial instant of time, and since $|1_A\rangle =\left( |0_1,0_2,1_3\rangle -|1_1,0_2,0_3\rangle \right) /\sqrt{2}$, we observe that the end cavities are equally populated $\left[ {\mathcal {P}}^{( 1_1)} (0)={\mathcal {P}}^{( 1_3)} (0)=1/2\right]$, while the central cavity is empty $\left[ {\mathcal {P}}^{( 1_2)} (0)=0\right]$. As time evolves, the excitation is progressively transferred to the two qubits at the same time, until the maximum simultaneous qubit excitation [with ${\mathcal {P}}^{( ee)} (0)=1$] is reached at $t=\pi /(2 \Omega _{{\mathrm{eff}}})$. It is interesting to observe that, since we are exciting the antisymmetric mode, the central cavity remains empty during the whole process. This fact remains valid even if the central cavity is detuned $\left( \Delta \ne 0\right)$, so that the system displays the same dynamics in the non-zero detuning case, the only difference being that the simultaneous excitation of the two qubits is achieved at a different instant of time.

The excitation of the antisymmetric normal mode, which is the most effective way to achieve the desired effect, can be experimentally achieved by sending a suitable narrow Gaussian pulse to the first cavity, whose central frequency of the pulse has to be chosen to be in the middle of the two split transition energies $\omega _d=(\omega _{40}+\omega _{50})/2$. For the sake of simplicity, here we do not present numerical calculations for the dynamics of the system excited by a Gaussian pulse, since the results would not add any additional physical information.

#### Dynamics after exciting the first cavity

We now turn to the study of the system dynamics when, instead of exciting the antisymmetric mode, we directly excite only the first cavity. Unlike the previous case, this process strongly depends on the detuning $\Delta$.

The dynamics of the occupation probabilities ${\mathcal {P}}^{(k)}$ for a three coupled cavity–qubit array system initially prepared in the one-photon state $|1_1\rangle$ can be studied for arbitrary values of the detuning $\Delta$ by using a semi analytical approach. Choosing the energy eigenstates $|E_j\rangle$ of $\hat{{\mathcal {H}}}^{\prime }$ as basis, and considering that only the states $|E_3\rangle ,|E_4\rangle ,|E_5\rangle$, and $|E_6\rangle$ are involved in the process, the time evolution of the initial state $|1_1(t)\rangle$ in the Schrödinger picture can be written as:

21 $$\begin{aligned} |1_1(t)\rangle =\sum \limits _{j=3}^{6} c_{j}\,e^{-i \omega _{j}t}|E_j\rangle , \end{aligned}$$

where $\omega _{j}$ are the numerically evaluated eigenvalues and the coefficients $c_{j}$, which depend on *J* and $\Delta$, are given by the elements of the transformation matrix in Eq. (18). The time evolution of the occupation probability ${\mathcal {P}}^{(k)}(t)$ for a generic state $|k\rangle =\sum \limits _{j=3}^{6} d_{j}|E_j\rangle$ are simply given by:

22 $$\begin{aligned} {\mathcal {P}}^{(k)}(t)=\left| \langle k|1_1(t)\rangle \right| ^2 = \left| \sum \limits _{j=3}^{6} c_{j} d_{j} \,e^{-i \omega _{j}t} \right| ^2. \end{aligned}$$

Figure 7b shows the time evolution of the occupation probabilities ${\mathcal {P}}^{( 1_1)} (t)$, ${\mathcal {P}}^{( 1_2)} (t)$, ${\mathcal {P}}^{( 1_3)} (t)$, and ${\mathcal {P}}^{( ee)} (t)$ for the resonant case $\Delta =0$, when the system is initially prepared in the one-photon state

23 $$\begin{aligned} |\psi _1\rangle =\left( |0_{\text {S}_1},1_{\text {S}_2},0_A,g,g\rangle -|1_{\text {S}_1},0_{\text {S}_2},0_A,g,g\rangle \right) /2 -|0_{\text {S}_1},0_{\text {S}_2},1_A,g,g\rangle /\sqrt{2} =|1_1\rangle . \end{aligned}$$

As the time evolves, we observe that the excitation, continuously propagating between the three cavities, is progressively transferred to both qubits at the same time, even if they are detuned from the central cavity. We observe that initially the system dynamics is not affected by the presence of the qubits and the system behaves like an array of three weakly-coupled resonant cavities. Once the excitation starts to be transferred to the qubits, the system undergoes a more complex dynamics and the probability for the qubits to be simultaneously excited reaches its maximum ${\mathcal {P}}^{( ee)}=1/2$ at $t=\pi / (2 \Omega _{{\mathrm{eff }}})$. As expected, at this instant of time the electric field is completely delocalized only in the end cavities with ${\mathcal {P}}^{(1_1)}={\mathcal {P}}^{( 1_3)}=1/4$, while the central cavity is depopulated. This result can be easily understood by considering that, after the system is prepared in the state $|\psi _1\rangle$, the superposition $\left( |0_{\text {S}_1},1_{\text {S}_2},0_A,g,g\rangle -|1_{\text {S}_1},0_{\text {S}_2},0_A,g,g\rangle \right) /2$ evolves freely since the qubits are not coupled to the symmetric modes.

In contrast to this, due to the interaction between the qubits and the antisymmetric mode, the above-described coherent-energy-exchange process $|0_{\text {S}_1},0_{\text {S}_2},1_A,g,g\rangle \rightarrow |0_{\text {S}_1},0_{\text {S}_2},0_A,e,e\rangle$ takes place so that at $t=\pi / (2 \Omega _{\mathrm{eff }})$ the system will be in the state

24 $$\begin{aligned} |\psi _2\rangle =\left( |0_{\text {S}_1},1_{\text {S}_2},0_A,g,g\rangle -|1_{\text {S}_1},0_{\text {S}_2},0_A,g,g\rangle \right) /2 -|0_{\text {S}_1},0_{\text {S}_2},0_A,e,e\rangle /\sqrt{2}. \end{aligned}$$

The observed values for the occupation probabilities ${\mathcal {P}}^{(k)}$ in Fig. 4b can be explained by considering that, in terms of the energy eigenstates of the Hamiltonian of the uncoupled system $(J=g=0)$, this state can be expressed as

25 $$\begin{aligned} |\psi _2\rangle =\left( |1_{1},0_{2},0_3,g,g\rangle +|0_{1},0_{2},1_3,g,g\rangle \right) /2 -|0_1,0_2,0_3,e,e\rangle /\sqrt{2}. \end{aligned}$$

Finally, in Fig. 7c we present numerical results for the dynamics of the system initially prepared in the state $|\psi _1\rangle$ for $\Delta /\omega _c=0.5$. In this case, the excitation of the first cavity can be obtained by exciting a superposition of the lowest-energy symmetric mode and the antisymmetric mode (see Fig. 5b). This could be experimentally realized by sending a suitable broad Gaussian pulse to the first cavity, able to excite the energy levels $|E_{3}\rangle$, $|E_{4}\rangle$, $|E_{5}\rangle$ and $|E_{6}\rangle$ simultaneously.

We observe that the system dynamics displays a different trend with respect to the resonant case. Indeed, due to the strong detuning, the central cavity acts like a high-potential barrier and the excitation is transferred back and forth via photon tunneling only between the end cavities, with the system effectively behaving like a two coupled cavity–qubit array. During the whole process, the central cavity remains very low-populated and the excitation is progressively transferred to the qubits, and the maximum simultaneous excitation probability ${\mathcal {P}}^{( ee)}=1/2$ is reached at $t=\pi / (2 \Omega _{\mathrm{eff }})$, where $\Omega _{{\mathrm{eff }}}/\omega _{c}= 4.5 \times 10^{-4}$ is the effective coupling for $\Delta /\omega _c=0.5$ between two qubits and a single photon.

The processes described here could be experimentally observed by placing two superconducting artificial atoms at opposite ends of an array of capacitively-coupled superconducting waveguides. These anomalous multiatom excitation and emission processes can find applications for the development of novel quantum technologies for quantum information and communication as, for example, the realization of new effective methods for quantum information transfer between photons and qubits in quantum networks.

## Conclusions

When the light–matter coupling strength increases, the vacuum fluctuations of the electromagnetic field become able to efficiently induce virtual transitions, replacing the role of the intense applied fields in nonlinear optics^10,11,81^. In this way, higher-order processes involving counter-rotating terms can create an effective coupling between two states of a system with different numbers of excitations. One of the most interesting examples is the process where a single photon in an electromagnetic resonator can jointly excite two atoms interacting with the same resonator. We have investigated this intriguing nonlinear optical process in the case where each of the two atoms is coupled to a distinct resonator. Specifically we studied: (1) the case of two coupled resonators, each of them interacting with a single atom, and (2) the case of two resonator-atom systems weakly coupled through a central resonator (resonant or detuned with respect to the other two). We studied the dynamics of these systems under different excitation conditions, showing that a coherent energy transfer between a single photon and two spatially separated atoms can still occur with a probability approaching one, under specific excitation conditions.

The results obtained provide interesting insights into subtle causality issues underlying the simultaneous excitation of two-level systems placed in spatially separated resonators. The processes described here could be experimentally realized in state-of-the-art circuit QED systems. Specifically, in our calculations we used a normalized coupling strength $\eta =|g|/\omega _c = 0.3$. It is not a problem to experimentally reach such a value in circuit QED systems. Using a flux qubit coupled to an *LC* oscillator, values of $\eta$ of the order of $\simeq 0.65$, and even beyond it, have been obtained. In order to observe the dynamics shown, e.g., in Fig. 3, the broadenings should be significantly lower than the splitting shown in Fig. 2b. Such a splitting is of the order $\Delta / \omega _c \sim 10^{-3}$. For a typical resonance frequency $\omega _c / 2 \pi \simeq 6$ GHz, the splitting is thus of the order of 0.35 GHz. In circuit QED systems, typical damping rates are orders of magnitude lower than this value^5^. Notice also that such a splitting can be increased by increasing $\eta$; it scales as $\eta ^3$^23^. Recently, the possibility to coherently couple distant ($\sim 1$ m) cavity-QED systems, connecting them by a microwave cable with negligible loss^88,89^ has been experimentally demonstrated. These results opens the possibility to implement the process proposed here involving distant artificial atoms.

It would be interesting and useful for applications to extend these studies to the cases of spatially separated atomic ensembles^86^, and distant qubits coupled to open waveguides^93^. Moreover, further insights on the processes here analysed can be gained by considering two or more spatially separated atoms in a multimode cavity^87^. It would also be interesting to extend the description of other vacuum-boosted nonlinear optical processes in the USC regime^25,81^, including coupled and/or multi-mode resonators.
